# Assessment of medicines cold chain storage conformity with the requirements of the World Health Organization in health facilities of the Eastern Province of Rwanda

**DOI:** 10.1186/s40545-023-00534-3

**Published:** 2023-02-28

**Authors:** Joseph Désiré Nyirimanzi, Joseph Ngenzi, Védaste Kagisha, Thomas Bizimana, Egide Kayitare

**Affiliations:** 1 East Africa Community Regional Center of Excellence for Vaccines, Immunization and Health Supply Chain Management (RCE-VIHSCM), College of Medicine and Health Sciences, University of Rwanda, Kigali, Rwanda; 2 Department of Pharmacy, School of Medicine and Pharmacy, College of Medicine and Health Sciences, University of Rwanda, Kigali, Rwanda

**Keywords:** Storage temperature, Cold chain, Pharmaceutical products, Supply chain, Conformity

## Abstract

**Background:**

Despite the prominent evidence of cold chain monitoring in the health system to reduce wastage and maintain product potency, there are still inefficiencies in the storage and transportation of cold chain products. This study assessed medicine cold chain storage conformity in health facilities in the Eastern Province of Rwanda.

**Methods:**

A cross-sectional and prospective with quantitative and qualitative approaches study was approved, and 44 health facilities (public, faith-based and private) were selected using both convenience, stratified, and purposive sampling techniques. Temperature data loggers were mounted in refrigerators to measure the Mean Kinetic Temperature (MKT).

**Results:**

The overall performance of cold chain storage conformity of refrigerators assessed was 54 (73.0%). The conformity found in refrigerators of pharmacy stock in public health facilities was 22 (56.0%), in vaccination program was 25 (100.0%), and in private retail pharmacies was 7 (70.0%). The MKT measured fit the required cold chain storage at this rate. Most refrigerators used in pharmacy stock (27.0%) were aged between 8 and 10 years, while those used in vaccination programs were less than 4 years. Regular calibration of refrigerator and temperature monitoring device (TMD) was 39.0% and 24.0%, respectively. Most respondents, 44 (80.0%), knew the proper cold chain storage. However, few identified the impact of storing cold chain products with vaccines at 16 (29.1%). The transportation of cold chain products from two central medical stores assessed showed inefficiencies as only one of them uses temperature data loggers during transportations of the products mentioned above.

**Conclusions:**

The medicines cold chain storage conformity investigated in seven districts of the Eastern Province in Rwanda was better than reported previously in other LMICs. However, cold chain storage for pharmacy stock often did not meet the requirements. The observed drawback represented a serious risk to public health. Calibrating cold chain equipment, regular maintenance, and commissioning new cold chain equipment should be done to improve cold chain storage.

## Background

Accessibility and affordability of vaccines and essential medicines are a preoccupation of the 2030 agenda for sustainable development goals (SDGs) to achieve universal health coverage [1]. On the other hand, cold chain product availability saves lives in terms of maternal mortality reduction and management of non-communicable diseases [2, 3]. Moreover, the intended beneficiaries reap reliable benefits from the robust cold chain management, notably healthcare service delivery and widened immunization coverage.

A cold chain is a temperature-controlled supply chain for products such as pharmaceuticals, vaccines, and blood components that require a specific temperature range during distribution and storage [4]. It requires a complex series of multiple links that involve various facilities, storage conditions, transportation modes, and trained personnel for accurate and proper management [5].

The efficiency of a cold chain system requires three main elements: a trained cold chain technician, reliable storage, and proper transportation facilities [6]. In addition, the cold chain monitoring system ensures proper cold chain functionalities, including temperature monitoring devices (thermometers, data loggers) and charts [7].

The unsuitable cold chain management poses a potential risk to the well-being of a patient, including loss of potency and ineffectiveness, culminating in contracting any disease due to a weak immune system [8]. The quality of cold chain products depends on the storage and handling strategies. These temperature-sensitive products should be handled under specific conditions predetermined by the manufacturer [9]. Thus, temperature fluctuations of the cold chain products should be managed under a comprehensive approach [10]. Monitoring the cold chain conformity is essential to maintain product quality and protect end-users against ineffective pharmaceutical products [11].

The World Health Organization (WHO) established guidelines for storing and transporting temperature-sensitive products. The aim was to set up harmonized standards of cold chain management of pharmaceuticals, ensuring the health products’ efficiency, quality, and efficacy, and detailed information on temperature-controlled transport [12].

The study carried out in Ethiopia pointed out the inefficiencies in storage practices of health commodities. Observed deviations include temperature recording, storage capacity, insufficient knowledge in cold chain handling, and lack of generators as backup systems during a power supply failure [13]. In Tanzania, 33 (48.5%) of assessed health facilities deviated from WHO recommendations in temperature monitoring [14]. The assessment of effective vaccine management carried out by WHO and the United Nations Children’s Fund (UNICEF) in 2014 in low and middle-income countries showed inefficiencies in vaccine management systems, including vaccine stock-outs, insufficient and un-functional cold chain equipment, and a lower rate of temperature monitoring system [15].

In Rwanda, the Expanded Program for Immunization (EPI) procures vaccines from WHO and UNICEF and distributes vaccinations through district hospitals and health centers [16]. On the other hand, Rwanda Medical Supply Ltd (RMS) procures essential medicines, including cold chain products from manufacturers and suppliers, and distributes them to service delivery points comprising district hospitals and health centers [17]. The private health facilities do not manage vaccines. Instead, procure other cold chain products from private pharmaceutical wholesalers.

As a result of global warming, climate change negatively affects supply chain operations during manufacture and storage, especially cold chain products stored in inappropriate conditions [18]. Rwanda is among the countries the climate change impacts its economies [19]. For example, the Eastern Province counts long dry spells characterized by rainfall deficit throughout the year. This drawback raises the temperature abnormally, which might affect cold chain storage in the case of a power outage [20]. In addition, according to the Long-Term Climate Risk Index (CRI) in 2017, Rwanda was ranked 130th among 180 countries with climate vulnerability [20]. Moreover, according to the Rwanda Demographic Health Survey (RDHS) of 2019–2020, only 43.0% of households in Eastern Province have access to electricity which may negatively impact the cold chain management systems [21].

This study assessed cold chain storage conformity to the World Health Organization standards in the Eastern Province of Rwanda.

## Methods

This study was conducted at public and private health facilities in the Eastern Province (EP) of Rwanda. The Eastern Province is divided into seven districts and covers a total area of 9813 km^2^. According to the national census of 2012, this is the most populated among Rwanda’s five provinces, estimated 2,595,703 inhabitants. Climatically, the EP is dominated by prolonged dry spells and drought with deficit rainfall which may raise temperature abnormally. A cross-sectional, prospective, and observational study was carried out using a quantitative and qualitative approach in selected health facilities (public, faith-based and private) in the Eastern Province of Rwanda from November 2021 to February 2022.

### Sample size and sampling techniques

The 210 health facilities were grouped into strata comprising public health centers (106), faith-based facilities (17), district hospitals (7), RMS branches (7), Central medical stores (2), and 71 private retail pharmacies (Table 1). We excluded private clinics as all of them do not manage cold chain products [22]. It has been convened to select two public health facilities and two faith-based health facilities in each of the seven districts comprising the Eastern Province of the above strata using the RAND Function of Microsoft Office Excel 2016 (Table 1). In addition, two central medical stores (EPI program and RMS head office), one hospital, and one RMS branch were also selected purposively, as they distribute cold chain products to the intermediate supply chain levels. One hospital and RMS branch in each district were also chosen purposively as they distribute cold chain products (vaccines and other temperature-sensitive medicines) to the service delivery points (SDPs) for health care delivery and immunization.

**Table 1:** Sample size

District	Health center	Faith-based facility	Hospital	Private pharmacy	RMS branch	Total
Bugesera	15	2	1	21	1	40
Gatsibo	17	3	1	7	1	29
Kayonza	12	4	1	10	1	28
Kirehe	19	0	1	2	1	23
Ngoma	11	4	1	5	1	22
Nyagatare	18	2	2	15	1	38
Rwamagana	14	1	1	11	1	28
RMS HQ + EPI	2					2
Total population	108	16	8	71	7	210
Sample size	16 (14 health centers + 2 central medical stores: RMS HQ + EPI)	14	7	14	7	58

Finally, two refrigerators were chosen in each health center, district hospital, and RMS branch for assessment (one for vaccination and one for storing other routine cold chain products). In private retail pharmacies, only one refrigerator was selected as they do not manage vaccines and manage very few cold chain products (Table 1).

Furthermore, two cold chain technicians were selected in each health center, district hospital, faith-based facilities, and RMS branches. Each central medical store and private retail pharmacy selected one cold chain technician. Hence, the 58 health facilities comprising 98 refrigerators and 93 cold chain technicians provided a sampling frame. Nevertheless, due to the movement restrictions imposed to constrain the COVID-19 pandemic outbreak, out of 58 health facilities, 44 (76.0%) were surveyed in this study. Moreover, 74 (76%) refrigerators and 67 (72.0%) cold chain technicians participated in this study.

As the public and faith-based health facilities manage almost all kinds of cold chain products, the knowledge on cold chain storage practices was only assessed among 55 technicians working in those categories of healthcare facilities.

Cronbach’s alpha (**α**) was pre-tested to ensure the reliability of the questions and found that *α* = 0.7, which indicates the reliability of the questions.

### Data collection method

Data were collected on health infrastructure, cold chain’ ‘technician’s knowledge, temperature monitoring system, quality management system, and the transportation policy of cold chain products in central medical stores of study areas. The closed-ended questionnaire was disseminated face-to-face to the respondent, and a checklist was used to collect relevant information aligned with study objectives.

In addition, temperature data loggers (Tempmate M1 Version 1.3 by imec Messtechnik GmbH, Heilbronn, Germany) were mounted in refrigerators and recorded the temperature fluctuations for 30 days every 10 min. These data loggers were connected to the computer with appropriate software to log selected parameters, including sampling intervals and start times. The imec Messtechnik GmbH software was used to calculate the Mean Kinetic Temperature (MKT) following the formula below:

$${\text{MTK}} = \frac{{\frac{\Delta H}{R}}}{{ - \ln \left( {\frac{{e^{{\left( {\frac{ - \Delta H}{{RT_{1} }}} \right)}} + e^{{\left( {\frac{ - \Delta H}{{RT_{2} }}} \right)}} + \ldots + e^{{\left( {\frac{ - \Delta H}{{RT_{n} }}} \right)}} }}{n}} \right)}},$$

where MTK is the mean kinetic temperature in kelvins; ∆*H* is the activation energy (in kJ mol^−1^); *R* is the universal gas constant (in J mol^−1^ K^−1^), i.e., 8.314472, *T* = temperature in degrees K; *n* = the number of sample periods over which data are collected; ln = is the natural log, and *e*^*x*^ is the natural log base.

### Data analysis

The Mean Kinetic Temperature (MKT) generated by the imec Messtechnik software was compared to the cold chain storage standards and pointed out deviations. The data on demographic profile, cold chain knowledge, storage, and infrastructure conditions were analyzed using Statistical Package for Social Science (SPSS V25). Descriptive and inferential statistics were performed to analyze critical variables, including Chi-square and relationship variables.

## Results

### Socio-demographic characteristics of the participants

The results showed no difference between the gender of the respondent as female was represented by 50.9% and male was 49.1% (*P* = 0.714). The majority, 67.2%, were nurses, and 82.1% of respondents held a University Bachelor’s degree. The experience distribution, age group, education level, and professional level were statistically significantly distributed (*P* < 0.005) in cold chain management. The majority, 47.3%, had not more than 3 years of experience, and only 14.5% had over 12 years of experience, as shown in Table 2.

**Table 2:** Socio-demographic characteristics of respondents

Variable	Category	Frequency	Percentage	Chi-square	*P* value
Gender	Male	35	52.2		
	Female	32	47.8	0.134	0.714
	Total	67	100.0		
Age group	20–30	22	**33.0**		
	31–40	34	**51.0**	11.851	0.003
	41 and above	11	**16.0**		
	Total	67	100.0		
Education level	Secondary	12	17.9		
	University	55	82.1	27.597	0.000
	Total	67	100.0		
	Pharmacist	20	**30.0**		
Profession	Nurse	45	**67.0**	41.761	0.000
	Clinical psychologist	2	**3.0**		
	Total	67	100.0		
	0–3 years	31	46.3		
Experience	4–7 years	13	19.4	17.716	0.001
	8–12 years	15	22.4		
	Above 12 years	8	11.9		
	Total	67	100.0		

### Cold chain storage facilities and their performance

Out of 74 refrigerators assessed, **50 (68.0%)** were used in public health facilities, 14 (19.0%) in faith-based facilities, and 10 (14.0%) used in private retail pharmacies. Most refrigerators, 49 (66.0%), were used to store cold chain products in pharmacy stock, including 25 used in public and faith-based health facilities, 14 used in Rwanda Medical Supply Ltd, and 10 used in private retail pharmacies. On the other hand, the vaccination programs covered 25 (34.0%) refrigerators used in public and faith-based facilities.

The conformity of cold chain storage of refrigerators used in pharmacy stock in public health facilities was 22 (56.0%) and in private retail pharmacies was 7 (70.0%).

Out of 39 refrigerators assessed in pharmacy stock of both public and the faith-based facility and RMS Ltd, 17 (44.0%) did not comply with the WHO standards of cold chain storage. The high MKT recorded was 21.4 °C, while the lowest was − 3.4 °C.

**Fig. 1:**
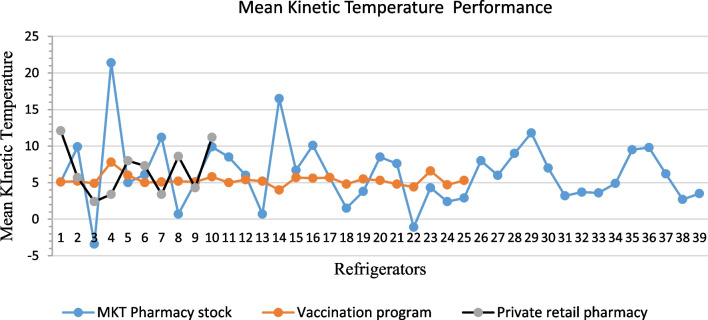
Compliance with the storage temperature with the WHO standards in different health facilities. The blue line indicates the Mean Kinetic Temperature (MKT) measured in Pharmacy stock. The orange line highlights the Mean Kinetic Temperature measured in the vaccination program, while the green line indicates the Mean Kinetic Temperature measured in private retail pharmacies. These MKT were caught after the application of data loggers Tempmate M1. In pharmacy stock, we assessed 30 refrigerators. In vaccination, we assessed 25 refrigerators, in private retail pharmacies, we assessed 10 refrigerators

However, 25 (100.0%) of refrigerators assessed in the vaccination program complied with cold chain storage World Health Organization (WHO) requirements. The highest MKT recorded in these refrigerators was 7.8 °C, while the lowest was 4 °C. Furthermore, out of 10 refrigerators assessed in private retail pharmacies, 3 (30.0%) did not comply with WHO requirements. The high MKT was 12.1 °C, while the lowest was 2.4 °C (Fig. 1).

**Fig. 2:**
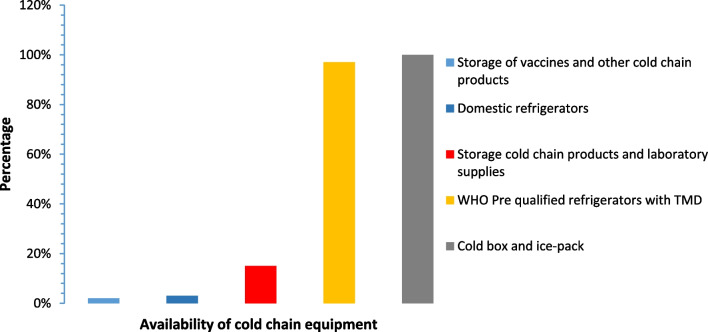
Availability of cold chain equipment. Vertical bars represent different cold chain equipment availability and usability

The overall performance of 74 refrigerators revealed that 54 (73.0%) refrigerators used in public, faith-based, and private retail pharmacies complied with the WHO cold chain storage standards. Among 74 refrigerators assessed, 64 (86.0%) were used to store cold chain products in public and faith-based health facilities, while 10 (14.0%) were used to store cold chain products in private retail pharmacies. In addition, 72 (97.0%) were WHO prequalified and furnished with temperature monitoring devices (thermometers or fridge tags). Availability of cold boxes and ice packs were found in all facilities (Fig. 2). However, 11 (17.0%) of refrigerators used in public and faith-based facilities are used to store various cold chain products, including cold chain medicines (oxytocin, insulins) and laboratory commodities (laboratory reagents, blood components, and other chemicals) in the same refrigerators. It was also found that 2 (3.0%) of public and faith-based health facilities used domestic refrigerators for storing cold chain products. One of the refrigerators assessed (1.0%) was used to store both vaccines and other cold chain products as one for vaccines was out of service (Fig. 2).

Among 64 refrigerators assessed in public and faith-based health facilities, 18 (28.0%) were aged between 2 and 4 years since their installation. They were used to store vaccines, while 17 (27.0%) and 9 (14.0%) were aged between 8 and 10 years and more than 10 years, respectively (Table 3). Out of 67 cold chain technicians interviewed, 61(91.0%) recorded daily storage temperature, whereas 6 (9.0%) did not record from many months ago. Out of 74 refrigerators assessed, only **25 (34.0%)** used thermometers and data loggers to monitor refrigerators’ temperature; all of these were used in the vaccination program. Nevertheless, 49 (66.0%) of refrigerators assessed use only thermometers to monitor the temperature status of refrigerators (Table 3). Regarding the contingency plan, out of 67 cold chain technicians assessed 53 (79.1%), confirmed using a generator in case of a power outage. Moreover, 8 (11.9%) wait for the upcoming power supply, and the remaining 6 (**8.9%**) use other means to maintain the temperature, such as solar energy, a cold box with a conditioned ice park, or other possible solutions, including storage at the nearest health facilities (Table 3). However, only 29 (43.3%) of the respondents confirmed having automated generators to cater for power failure after closing hours. Quality Management System (QMS) ensures that the cold chain equipment is calibrated and validated to maintain cold chain products appropriately in the cold chain management system. Out of the 67 cold chain technicians who participated in this study, 26 (39.0%) confirmed to have calibrated refrigerators once a year, 16 (24.0%) highlighted calibration of temperature monitoring devices. Moreover, among two central medical stores that distributes cold chain products only one confirmed to use a calibrated vehicle to distribute vaccines countrywide (Table 3).

**Table 3:** Parameters of temperature monitoring system

Parameter assessed	Area of operation	Category	Frequency	Percentage
Age of refrigerator	Vaccination program	2–4 years	18	28.0%
		< 2 years	7	11.0%
	Pharmacy stock	2–4 years	2	3.0%
		5–7 years	11	17.0%
		8–10 years	17	27.0%
		> 10 years	9	14.0%
Calibration of cold chain equipment	Pharmacy stock and vaccination	Refrigerator	26	39.0%
		Temperature monitoring device	16	24.0%
Temperature recording	Pharmacy stock and vaccination	Daily temperature recording	61	91.0%
		Use of thermometers only	49	66.0%
	Vaccination program	Use of data loggers and thermometers	25	39.0%
Contingency plan	Pharmacy stock and vaccination	Generator	53	79.1%
		Automated generator	29	43.3%
		Put vaccine in vaccine carriers	1	1.5%
		Solar energy	1	1.5%
		Store in other refrigerators	1	1.5%
		Use of ice pack	3	4.5%
		Waiting for the power supply to come back	8	11.9%

### Knowledge of cold chain technicians on cold chain storage practices from the public and faith-based health facilities

Technicians’ knowledge of cold chain maintenance was assessed through 14 Likert scale questions (Table 4). This assessment was done with cold chain technicians working in public and faith-based facilities as they managed vaccines and cold chain products. The questions focused on vaccine cold chain management and evaluated the VVM knowledge, vaccine storage, and distribution. The Likert scale table indicated that 27 (49.1%) cold chain technicians had a problem recognizing the shared responsibility of cold chain actors. The duration of vaccine storage at the district hospital is 1 month, and there was no difference between those who agreed and disagreed (*P* = 0.225). Vaccines storage between 2 and 8 °C was known by 44 (80.0%).

**Table 4:** Knowledge of cold chain storage good practices (*n* = 55)

The 14 Likert scale questions	Frequency (%)	Chi-square	*P* value
The vaccine cold chain maintenance is the responsibility of the hospital	28 (50.9)	30	0.000
The duration of keeping vaccines at the hospital is less than 2 months	23 (41.8)	7.891	0.225
All vaccines are stored between 2 and 8 °C	44 (80.0)	19.8	0.000
The VVM is used for vaccines damaged by heat	39 (70.9)	9.618	0.002
The VVM is used for vaccines damaged by freezing	19 (34.5)	5.255	0.022
A shake test must be conducted when freeze-sensitive vaccines are visibly seen to be frozen	30 (54.5)	0.455	0.500
Data loggers are used to measure temperature-sensitive medicines in cold chain storage	40 (72.7)	11.364	0.001
There are four stages of VVM identifying the state of vaccines	25 (45.5)	0.455	0.500
In the case of a shortage of refrigerators, vaccines and other temperature-sensitive medicines can be stored in the same refrigerator	16 (29.1)	9.618	0.002
Vaccines are distributed at − 2 and − 8 °C	18 (32.7)	6.564	0.010
Vaccines are transported using an ice pack in the cold box to maintain temperature	32 (58.2)	1.473	0.225
During outreach activities, vaccines should be stored in a vaccine carrier with ice pack	31 (56.4)	0.891	0.345
Before distributing vaccines, you should check only the expired date	10 (18.2)	22.273	0.000
Before distributing vaccines, you should check only their VVM	8 (14.5)	27.655	0.000

The majority of the respondent, 39 (70.9%), knew that VVM is used to measure if vaccines are damaged by heat, and 36 (65.5%) knew that VVM is not used to perform vaccine freezing. Nearly a half of the respondents, 25 (45.5%), identified the four VVM stages. Few respondents identified the impact of storing cold chain products with vaccines at 16 (29.1%). The knowledge of vaccine transportation and storage during outreach activities in the required storage conditions was significant as (*P* = 0.225) and (*P* = 0.345), respectively. Nevertheless, most respondents knew that before administering vaccines, it is important to check all requirements to ensure potency and safety, including expiry date **45 (81.8%)** and vaccine vial monitoring **47 (85.5%)**. Temperature data loggers were known as a tool used to record temperature for sensitive medicines in cold chain storage by **40 (72.7%)** respondents.

### Transportation of cold chain products from central medical stores to service delivery points

The cold chain management of health products was found to be parallel. RMS Ltd manages routine and vital cold products, while Expanded Program for Immunization manages vaccines and related supplies. The RMS ltd distributes cold chain products from the central medical store using the traditional vehicles. The **cold** chain products distributed are not controlled with temperature monitoring devices from the central medical stored to the intermediate cold chain levels (RMS branches) to monitor potential temperature variations. Moreover, RMS Ltd does not have a system to ensure the quality of cold chain products at the last mile during transportation.

In contrast, The Expanded Program for Immunization (EPI) had an electronic system that enabled the program to monitor cold chain equipment in remote areas and maintenance plans for equipment and refrigerated vehicles that distributed vaccines countrywide.

This facility also distributes vaccines using refrigerated and calibrated vehicles countrywide to ensure products reach the end-users with guaranteed safety and quality and disseminate temperature data loggers in each refrigerator to record temperature daily.

## Discussion

The attainment of universal health coverage across the globe is one of the ultimate goals of the 2030 agenda of sustainable development goals (SDGs). The key elements are improved quality, safe and effective medicine, and vaccines for everyone in need [1, 23]. In this regard, the availability of a well cold chain trained staff, storage conformity to the WHO standards, and suitable distribution and temperature monitoring equipment matter. In the current study, 20 (27.0%) of refrigerators did not conform to the WHO cold chain standards. The MKT calculated deviated the storage conformity as in some cases it was below 2 °C and in others above 8 °C. This result is slightly higher than the findings of the study conducted in Ghana. The better management of the cold chain in Ghana was attributed to the involvement of cold chain technicians [24]. However, our finding was significantly lower than the study in Tanzania 33 (46.5%) [25].

In contrast, it was observed that all refrigerators used in the immunization program complied with WHO cold chain requirements. The MKT calculated fit with the storage temperature requirements of the cold chain medicines range (2–8 °C). Good performance in the vaccination program might be due to the consistent use of WHO prequalified and newly commissioned refrigerators. In contrast, the lower performance of refrigerators used in pharmacy stock might be attributed to the oldness of refrigerators and the lack of a regular calibration system to maintain measurement capability and a contingency plan to maintain cold chain temperature.

In this study, the age of refrigerators used in pharmacy stock, since their commission was higher than those used in vaccination. These results are consistent with a study conducted in nine African countries and India on monitoring cold chain equipment. Their results revealed that many developing countries use obsolete technologies and old equipment and found that between 15.0 and 50.0% are susceptible to poor performance in temperature control and breakdown [26].

This study showed domestic refrigerators’ usability even at a minimum rate. These refrigerators are not recommended for cold chain product storage, because the temperature rises abnormally during defrosting [27]. Therefore, they cannot maintain the optimal temperature range [26]. Their use in different countries is due to their low cost [26].

The World Health Organization recommends calibrating temperature monitoring devices to ensure proven measurement accuracy [28]. The quality management approach of cold chain management pinpoints regular calibration of cold chain equipment by Standards Regulatory Bodies (SRB) to ensure a robust temperature monitoring system [29]. Our study showed inefficiencies in calibrating cold chain equipment for storage and transportation. This miss practice was also reported in African countries, such as Nigeria [30] and Ghana [31].

In this study, about 61 (91.0%) of respondents recorded temperature regularly, which is higher than the study conducted in Ethiopian health facilities [6] and Nigeria [30]. This difference might be due to different monitoring systems available in other countries.

Facilities used the generator to cater to the power outage. This result differs from the study conducted in Ghana [32]. However, these results are slightly higher than the study carried out in Nigeria [30], Ethiopia [13], and Tanzania [25]. These variances might be due to the extent health systems empower contingency plans of cold chain storage or financial capabilities. Although the availability of generators is high, almost half are not automated, which might impact the delay in starting the generator for various reasons in the case of a power outage.

One of our respondents supported these results and said: “our generator is not automated. In the case of electricity failure, if the trained staff is not around to start the generator, we remain in darkness, and refrigerators remain out of service till morning”. The respondent also pointed out the financial constraint for buying fuel, which impacts generator use in the case of a power supply outage.

The transportation of temperature-sensitive products, including cold chain products, requires special handling to ensure optimal quality, potency, and integrity from the manufacturer to the end-users [33]. The result of the study pointed out that the transportation of cold chain management also remains an issue in public health facilities, especially for routine cold chain products, as central medical does not control it throughout the distribution. In contrast, the EPI Rwanda program monitors vaccines’ transport until the end-users via refrigerated and calibrated vehicles, data loggers, and the cold chain report status of the equipment disseminated countrywide. The use of non-specialized cold chain transportation means was also reported in many other countries, such as Cameroon [13], Ethiopia [32], and Kenya [11].

### Study limitation

The study was conducted in one out of five provinces of Rwanda, and the duration of data collection was 1 month. Furthermore, it did not cover all factors associated with cold chain storage and distribution characteristics. The findings of this study were limited to the health facilities assessed in the Eastern Province of Rwanda, not generalized to the entire country.

## Conclusion

In this study, most refrigerators correctly displayed the conformity of cold chain storage towards the World Health Organization standards. Although most refrigerators have been old since their commission, most were connected to a backup system to cater to the power outage. A large proportion of refrigerators and temperature monitoring devices were not calibrated. Most respondents correctly knew the temperature range of storing cold chain medicine. Most respondents had insufficient information on the negative impact of keeping vaccines with other cold chain medicines. The transportation system of cold chain products from central medical stores to the service delivery points was limited due to the insufficient number of temperature monitoring devices.

The overall medicines cold chain storage conformity with the WHO requirements in seven investigated districts of the Eastern Province of Rwanda was better than the previous studies in other Low and Middle Incomes Countries (LMICs). However, cold chain storage for pharmacy stock often did not meet the requirements as the temperature storage of the refrigerators deviated from the WHO standards, and various contributing factors were pointed out. The non-conformity of cold chain storage in pharmacy stock may represent a serious risk to public health.

Validation of cold chain storage infrastructures through calibration of cold chain equipment, regular maintenance, and strategizing commissioning new cold chain infrastructures to mitigate the non-conformity observed resulting from shortcomings in cold chain monitoring should be done to minimize potential public health risks.

## Data Availability

The data sets used in this study are available from the corresponding author on a reasonable request.

## Abbreviations

CMHS: College of Medicines and Health Sciences
EPI: Expanded program on immunization
GAVI: Global alliance for vaccines and immunization
IRB: Institutional Review Board
LMICs: Low and Middle Incomes Countries
MKT: Mean Kinetic Temperature
RSB: Rwanda Standards Board
RMS: Rwanda Medical Supply Ltd
SDP: Service delivery point
TMD: Temperature monitoring device
UNICEF: United Nations Children’s Fund
VVM: Vaccine vial monitor
WHO: World Health Organization

## References

[1] United Nations. The 2030 agenda and the sustainable development goals an opportunity for Latin America and the Caribbean thank you for your interest in this ECLAC publication. 2018.

[2] Heinemann L, Braune K, Carter A, Zayani A, Krämer LA (2021). Insulin storage : a critical reappraisal. J Diabetes Sci Technol.

[3] Bizimana T, Hagen N, Gnegel G, Kayumba PC, Id LH. Quality of oxytocin and misoprostol in health facilities of Rwanda. PLoS ONE. 2021. 10.1371/journal.pone.0245054. 10.1371/journal.pone.0245054 PMC7793248 33417602

[4] Philippine Department of Health. Vaccines, cold chain and logistics management. Manual of operations for National Immunization Program. 2018;5. https://doh.gov.ph/sites/default/files/publications/NIP-MOP-Booklet%2010.pdf.

[5] Ontario College of pharmacists. Protecting cold chain. Pharm connect. 2012;19. https://www.ocpinfo.com/wpcontent/uploads/documents/Protecting_the_Cold_Chain_Part_I.pdf.

[6] Fekadu G, Merga G, Gebre M (2018). Assessment of medicines cold chain storage conformity with the national requirements in governmental health care facilities of Nekemte Town, Western Ethiopia. J Bioanal Biomed.

[7] Ogboghodo EO, Omuemu VO, Odijie O, Odaman OJ (2017). Cold chain management practices of health care workers in primary health care facilities in Southern Nigeria. Pan Afr Med J.

[8] World Health Organization [WHO]. If you chose not to vaccinate your child, understand the risks and responsibilities. CDC. 2016;1–3.

[9] Shastri D. Vaccine Storage and Handling. Textb Pediatr Infect Dis. 2018;493–493. Available at https://www.health.gov.on.ca/en/pro/programs/publichealth/oph_standards/docs/protocols_guidelines/Vaccine_Storage_and_Handling_Protocol_2018_en.pdf.

[10] Kumar N, Jha A (2017). Temperature excursion management: a novel approach of quality system in pharmaceutical industry. Saudi Pharm J.

[11] Njuguna MW, Mairura CJ, Ombui K (2015). Influence of cold chain supply logistics on the safety of vaccines. A case of pharmaceutical distributors in Nairobi County. Int J Sci Res Publ.

[12] World Health Organization. Annex 9: model guidance for the storage and transport of time and temperature-sensitive pharmaceutical products. WHO Tech Rep Ser No. 961, 2011. 2011. p. 324–72.

[13] Bogale HA, Amhare AF, Bogale AA (2019). Assessment of factors affecting vaccine cold chain management practice in public health institutions in East Gojam zone of Amhara region. BMC Public Health.

[14] Ringo S, Mugoyela V, Kaale E, Sempombe J (2017). Cold chain medicines storage temperature conformity by the World Health Organisation in Tanzania. Pharmacol Pharm.

[15] UNICEF, WHO. Achieving immunization targets with the comprehensive effective vaccine management (EVM) framework. WHO/UNICEF Jt statement. 2016;2014:1–5. Available from: http://www.who.int/immunization/programmes_systems/supply_chain/EVM-JS_final.pdf.

[16] Rwanda Biomedical Center. Comprehensive multi-year plan 2013–2017. 2012. p. 1–60. Available from: https://extranet.who.int/countryplanningcycles/sites/default/files/country_docs/Rwanda/attachment_6_revised_cmyp_08.pdf.

[17] Rwanda Ministry of Health. Health supply chain management module for RMS branches and central level Republic of Rwanda Ministry of Health. 2021.

[18] Dasaklis TK, Pappis CP (2013). Supply chain management in view of climate change: an overview of possible impacts and the road ahead. J Ind Eng Manag.

[19] Gaspard R, Tuyishimire J, Mugabowindekwe M, Mugisha J (2019). Assessing the impact of climate change and variability on wetland maize production and the implication on food security in the highlands and central plateaus of Rwanda. Ghana J Geogr.

[20] Rwanda Environment Management Authority (REMA), IUCN, Fund GC. Transforming Eastern Province of Rwanda’s capacity to adapt to climate change through forests and landscapes restoration. 2019. p. 19.

[21] National Institute of Statistics of Rwanda. Rwanda demographic and health survey 2019–20 District Pro le East Province. 2019.

[22] Ministry of Health - Rwanda. Private health facilities in Rwanda health service packages. 2017.

[23] United Nations. The sustainable development goals report. 2021.

[24] Burstein R, Dansereau EA, Conner RO, Decenso BM, Delwiche KP, Gasasira A, et al. Assessing vaccine cold chain storage quality: a cross-sectional study of health facilities in three African countries. Lancet. 2013;381:S25. 10.1016/S0140-6736(13)61279-9.

[25] Ringo S, Mugoyela V, Kaale E, Sempombe J (2017). Cold chain medicines storage temperature conformity with the World Health Organisation requirements in health facilities in Tanzania. Pharmacol Pharm.

[26] Ashok A, Brison M, LeTallec Y (2017). Improving cold chain systems: challenges and solutions. Vaccine.

[27] Western Australia Departement of Health. Health Sate Use of Medication Refrigerators policy.2015: available at https://handsoninfectioncontrol.com.au/wp-content/uploads/2015/01/OD-0587-15-WA-Health-Safe-Use-of-Medication-Refrigerators-Policy.pdf.

[28] Vaccine WHO, Handbook M. How to monitor temperatures. 2015.

[29] Botting A, Agreement L. AS/NZS ISO 9001:2008 quality management systems-Requirements. 2011.

[30] Chukwu OA, Adibe M (2022). Quality assessment of cold chain storage facilities for regulatory and quality management compliance in a developing country context. Int J Health Plann Manage.

[31] Adomako P. Public health challenges in the supply chain management. Master’s Thesis. 2012.

[32] Asamoah A, Innocentia N, Enyan E, Diji AK, Domfeh C (2021). Cold chain management by healthcare providers at a district in Ghana: a mixed methods study. Hindawi BioMed Res Int.

[33] Lebanese Ministry of Health. Good Cold Chain Management for Pharmaceutical Products. 2017. https://www.moph.gov.lb/userfiles/files/HealthCareSystem/Pharmaceuticals/QualityAssuranceofPharmaceuticalProducts/GoodColdChainManagementforTemperature-SensitivePharmaceuticalProducts-Edition2-2017.pdf.

[34] Yakum MN, Ateudjieu J, Walter EA, Watcho P (2015). Vaccine storage and cold chain monitoring in the North West region of Cameroon: a cross-sectional study. BMC Res Notes.

